# Eosinophils in skin diseases

**DOI:** 10.1007/s00281-021-00868-7

**Published:** 2021-06-07

**Authors:** Susanne Radonjic-Hoesli, Marie-Charlotte Brüggen, Laurence Feldmeyer, Hans-Uwe Simon, Dagmar Simon

**Affiliations:** 1 Department of Dermatology, Inselspital, Bern University Hospital, University of Bern, Bern, Switzerland; 2 Faculty of Medicine, University of Zurich, Zurich, Switzerland; 3 Department of Dermatology, University Hospital Zurich, Zurich, Switzerland; 4 Department of Dermatology, Hochgebirgsklinik Davos, Davos, Switzerland; 5 Institute of Pharmacology, University of Bern, Bern, Switzerland; 6 Department of Clinical Immunology and Allergology, Sechenov University, Moscow, Russia; 7 Laboratory of Molecular Immunology, Institute of Fundamental Medicine and Biology, Kazan Federal University, Kazan, Russia

**Keywords:** Eosinophil, Granule proteins, Host defense, Immunoregulation, Tissue damage

## Abstract

Eosinophil infiltration is a common finding in a broad spectrum of skin diseases, despite the fact that the skin is devoid of eosinophils under physiologic conditions. Although cutaneous eosinophilia is reactive, cytokine-mediated in most cases, diseases with an intrinsic mutation-mediated clonal expansion of eosinophils can also manifest on the skin. As eosinophils are involved in host defense, regulate immune responses, generate pruritus, induce remodeling and fibrosis, and can cause tissue damage, they have the capacity to actively contribute to the pathogenesis of diseases. Recent research provided deeper insights in the mechanisms, e.g., bacterial and viral clearance, blister formation, recruitment of cytotoxic T cells, and generation of pruritus, by which eosinophils might come into action. This review aims at providing an overview on the clinical presentations of eosinophil-associated dermatoses and the current understanding of their pathogenic role in these diseases. Further, we discuss the effects of therapies targeting eosinophils.

## Introduction

Eosinophil infiltration of the skin is a frequent histopathological finding in a broad spectrum of dermatological disorders. This observation might be astonishing considering the fact that the skin does not harbor eosinophils under physiologic conditions. As eosinophilic dermatoses lack any specific clinical sign or pattern, a skin biopsy and histology together with the clinical manifestations are often required to get the correct diagnosis. Under the microscope, eosinophils easily attract attention as they appear as bright red granular cells in hematoxylin and eosin (H&E) stained tissue. Indeed, the affinity to the red dye eosin was the reason why Paul Ehrlich named his newly discovered cells eosinophils [1, 2]. Eosinophilic dermatoses present with or without accompanying peripheral blood eosinophilia, defined as absolute eosinophil counts >0.5 G/l [3]. The development of targeted antieosinophil therapies that effectively deplete eosinophils in the blood and tissues has tremendously accelerated research on eosinophils in order to understand their function under physiologic and pathologic conditions. In the field of dermatology, progress has been made in demonstrating subgroups of eosinophils by their cytokine expression [4], different ways of mediator release [5], eosinophil-mediated effects on blister formation in bullous pemphigoid (BP) [6], vasopermeability [7], bacterial killing [8], and pruritus [9, 10], in elucidating eosinophil activation factors [8, 11], and roles in tumor biology [12, 13]. Moreover, antibodies affecting eosinophils have been demonstrated to be effective in improving cutaneous manifestations of hypereosinophilic syndromes [14, 15].

In this review, we are aiming at demonstrating the spectrum of eosinophilic diseases affecting the skin with special focus on the pathogenic roles of eosinophils and eosinophils as therapeutic targets.

## Eosinophils

As innate immune cells, eosinophils are involved in host defense. They express several pattern recognition receptors, including toll-like receptors (TLR), nucleotide-binding oligomerization domain-like receptors (NLRs), G protein-coupled, Fc, chemokine, adhesion, and cytokine receptors [16, 17]. Receptor stimulation leads to degranulation of toxic granule proteins (eosinophil peroxidase (EPO), eosinophil cationic protein (ECP), eosinophil-derived neurotoxin (EDN), and major basic protein (MBP)), synthesis of nitric oxide, release of cytokines, and chemokines, and cell trafficking [16, 17].

### Toxic granule proteins

Eosinophils are stuffed with granules, primary and secondary ones. Primary granules harbor Charcot-Leyden crystal (CLC) protein (galectin-10) and eosinophil peroxidase (EPO). A role of CLC has been associated with various eosinophilic including allergic and parasitic diseases. CLC is assumed to be involved in vesicular transport of cationic RNases and the granule formation during eosinophil differentiation [18]. EPO has been shown to exert antiparasitic and antibacterial effects. It is released either into large cytoplasmic vacuoles (phagosomes) or extracellularly where it binds to the surface of a target [18].

Secondary granules contain major basic protein (MBP) in the crystalline core, as well as eosinophil cationic protein (ECP), eosinophil-derived neurotoxin (EDN), and EPO in their matrix. ECP was shown to directly damage schistosomula of *Schistosoma mansoni* [19]. ECP, EDN and MBP have antibacterial properties that are dependent or independent of RNase activity [20–22]. Eosinophil-derived neurotoxin (EDN) is a member of the RNase superfamily. In addition to its ribonuclease activity resulting in neurotoxic and antiviral effects, EDN serves as a chemoattractant of dendritic cells, and as an endogenous ligand for toll-like receptor (TLR)2 [23]. ECP, MBP, and EPO have been identified as major components of eosinophil extracellular traps (EET) [5, 24]. To note, in the process of EET formation, degranulation and mitochondrial DNA release occur sequentially, suggesting that the DNA scaffold and granule proteins associate in the extracellular space [25]. In the granules, MBP is stored as nanocrystals that are nontoxic [26]. Its toxicity is triggered by granule acidification upon stimulation of eosinophils and extracellular aggregation [26]. Interestingly, further aggregation of MBP results in large amyloid plaques that are nontoxic and most likely limit tissue damage. Such MBP+ amyloid plaques have been observed in biopsies from eosinophilic cellulitis and atopic dermatitis (AD) [26].

### Mediator release

**Fig. 1:**
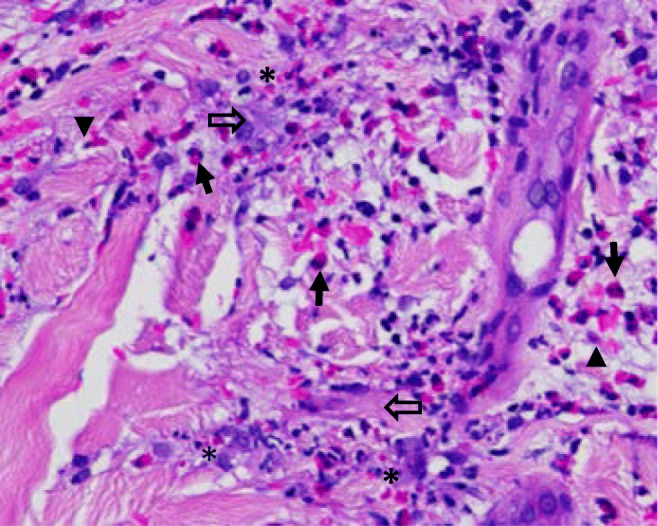
Eosinophil infiltration in the skin. The image shows round shaped eosinophils with their typical bi-lobed nuclei (arrow), cell-free granules indicating degranulation (*), cytolytic eosinophils (arrowhead), and flame figures presenting necrobiotic collagen fibres covered by eosinophil granule proteins (open arrow). Magnification ×400

Eosinophil function is dependent on the release of toxic granule proteins, cytokines, and chemokines (Fig. 1). These mediators are mostly stored within the secondary granules of eosinophils [27]. Different modes of degranulation exist, determining the execution of eosinophil function. In piecemeal degranulation, the most abundant form of degranulation found in tissue sections, eosinophils are capable of selectively secreting granule contents depending on the stimulus. For example, stimulation of eosinophils with RANTES or eotaxin-1 results in secretion of interleukin (IL)-4, whereas upon interferon (INF)-γ stimulation, eosinophils secrete IL-12 but not IL-4 [28]. On an ultrastructural level, piecemeal degranulation is characterized by a loss of density of secondary granules and the presence of so-called sombrero vesicles, corresponding to tubular structures serving as secretory vesicles, finally fusing with the plasma membrane for mediator release [28]. Classical exocytosis has been observed in close proximity to large parasites [29].

Eosinophil cytolysis, the second most common form of granule protein release into tissues, is the result of utmost activation of the eosinophil that results in cell death. On an ultrastructural level, chromatolysis and loss of plasma membrane integrity are found, alongside with deposits of so-called clusters of free eosinophil granules (cfegs). Interestingly, these partially intact cell-free granules appear to maintain effector function. Stimulation of cfegs with IL-4, RANTES or IFN-γ resulted in stimulus-dependent mediator release [30, 31]. Recently, adhesion-induced cytolysis was characterized as a nonapoptotic form of cell death with features of necroptosis, counter-regulated by autophagy [32]. Cytolysis-associated morphologic changes of eosinophils such as cytoplasmic vacuolization have been observed in various dermatoses [32].

Catapult-like release of mitochondrial DNA that associates with eosinophil granule proteins, resulting in the formation of net-like structures, is referred to as EET formation [24]. Extracellular traps are formed by viable cells, therefore, cell death, including eosinophil cytolysis, is not required [33]. Through EET formation, eosinophils are able to bind and kill extracellular bacteria [8]. EETs have been detected in a variety of skin disorders, including infectious, but also allergic and autoimmune diseases [5, 34].

### Cytokines expression by eosinophils

By the production and release of cytokines and chemokines, eosinophils function as immunomodulatory cells. They are often associated with type 2 inflammation as they respond to IL-5 and secrete cytokine such as IL-4, IL-9, and IL-13 that may affect Th2 cells, B cells, and macrophages [17]. By releasing GM-CSF, IL-8, and IL-10, eosinophils have the potential of attracting neutrophils, autostimulation, and immunoregulation [17]. Via synthesis of tumor necrosis factor (TNF)-α, eosinophils may exert cytotoxic effects [35]. To note, eosinophils are a source of IL-31, a cytokine that bridges inflammation and pruritus [9]. There is evidence that, with respect to cytokine production, distinct subpopulations of eosinophils exist in the skin depending on the underlying disease and inflammatory milieu [4].

### Fibrosis

Eosinophils have been reported to express CCL11 (eotaxin-1), tumor growth factor (TGF)-β, IL-6, IL-11, IL-13, and MMP-9 and thus may directly or indirectly stimulate tissue remodeling or fibrosis. A correlation of tissue eosinophils and mediator release with deposition of extracellular matrix proteins has been shown in skin diseases [4], eosinophilic esophagitis [36], and asthma [37]. TGF-β, which can either be released by eosinophils or by epithelial cells upon stimulation with IL-4 and IL-13, was shown to activate fibroblasts resulting in the secretion of matrix proteins, as well as the release of eotaxin stimulating an additional recruitment of eosinophils [38].CCL11 has profibrogenic properties on fibroblasts expressing its receptor CCR-3 [39]. In mouse models, IL-11 that is produced by eosinophils and structural cells, was shown to stimulate fibrosis [40].

## Potential roles of eosinophils in skin diseases

For most eosinophilic dermatoses, the exact pathogenic mechanism, specifically the role of eosinophils is still obscure. Here, we will discuss some general mechanisms together with the possible contribution of eosinophils.

### Host defense

Eosinophils are assumed to be involved in killing helminths particularly those in tissue-migratory stages by releasing toxic granule proteins and reactive oxygen species, as well as mediating protection against reinfection [41]. Notably, released eosinophil granule proteins cause concomitant tissue damage contributing to the disease pathology [41]. Antibacterial effects by eosinophils have been attributed to toxic effects of MBP and eosinophil cationic protein (ECP), the phagocytic capacity of eosinophils, although this is much less compared to neutrophils, and the formation of EETs [24]. Eosinophils have also been shown to promote virus clearance by releasing EDN, an RNase, that reduces virus infectivity, as well as activates dendritic cells by interacting with TLR2 resulting in IL-6 and TNF-α release, and functions as chemoattractant for macrophages [42]. Furthermore, eosinophils may function as antigen-presenting cells and stimulate CD8+ cells [43].

### Edema and blister formation

Eosinophils may contribute to edema by releasing eosinophil granule proteins, as well as by the production of leukotrienes that have direct vasodilatory effects on blood vessels or indirectly via stimulation of mast cells and basophils [44, 45]. Eosinophil infiltration in the skin and lining along the dermal-epidermal junction are observed in bullous pemphigoid (BP), an autoimmune-bullous disease. In the presence of BP autoantibodies eosinophils upon activation by IL-5 were shown to directly contribute to blister formation using an ex vivo skin model [6]. Adhesion, Fcγ receptor activation, elevated ROS production, and EET formation by eosinophils have been shown to be involved in dermal-epidermal separation [6]. Furthermore, ECP and EDN have been reported to exert cytotoxic effects on keratinocytes and cause cell-matrix detachment [46].

### Recruitment and activation of cytotoxic T cells

Through the recruitment and activation of cytotoxic T cells and natural killer (NK) cells, eosinophils have been demonstrated to restrict tumor growth [47–49], defend viral infection [43], and, likely, are involved in drug rash with eosinophilia and systemic symptoms (DRESS) [50].

### Induction of pruritus

Most eosinophilic dermatoses are associated with pruritus that can be incredible, e.g., in atopic dermatitis, prurigo nodularis and scabies. Eosinophils may stimulate nerve cells and contribute to pruritus by releasing granule proteins (ECP, EDN, MCP), mediators such as substance P, vasoactive intestinal peptide, brain-derived neurotrophic factor, neurotrophin-3, nerve growth factor, and cytokines such as IL-4, IL-13, and IL-31 [51]. A key role of IL-31 in causing itch has been demonstrated for atopic dermatitis, prurigo nodularis, dermatomyositis, and, with a direct contribution of eosinophils, for BP [10, 52–54]. In cutaneous T cell lymphoma (CTCL), eosinophil infiltration correlates with itch severity [55].

## Primary and secondary causes of eosinophilia

Since the clinical presentation of eosinophilic dermatoses is manifold and usually lacks specific criteria, a skin biopsy is indispensable to establish the correct diagnosis by histological, immunohistological and molecular analyses that should be complemented by blood tests and, if required, imaging. The accumulation of eosinophils in the skin and/or blood and other organs can be caused by either a mutation or gene fusion-mediated clonal expansion of eosinophils or a cytokine-mediated increased differentiation and survival of eosinophils. Intrinsic (clonal, neoplastic) eosinophilia is due to genetic changes in hematopoietic stem cells leading to chronic myeloid leukemias with eosinophils as part of the clonally expanded cells. Extrinsic, reactive forms of eosinophilia are the most common ones caused by cytokines such as IL-3, IL-5, and GM-CSF that are released by mainly T cells, as well as tumor cells [3, 56]. Determining the cause of eosinophilia has immense implications on the therapy of the underlying disease [57–59].

## Clinical patterns of eosinophilic dermatoses

**Fig. 2:**
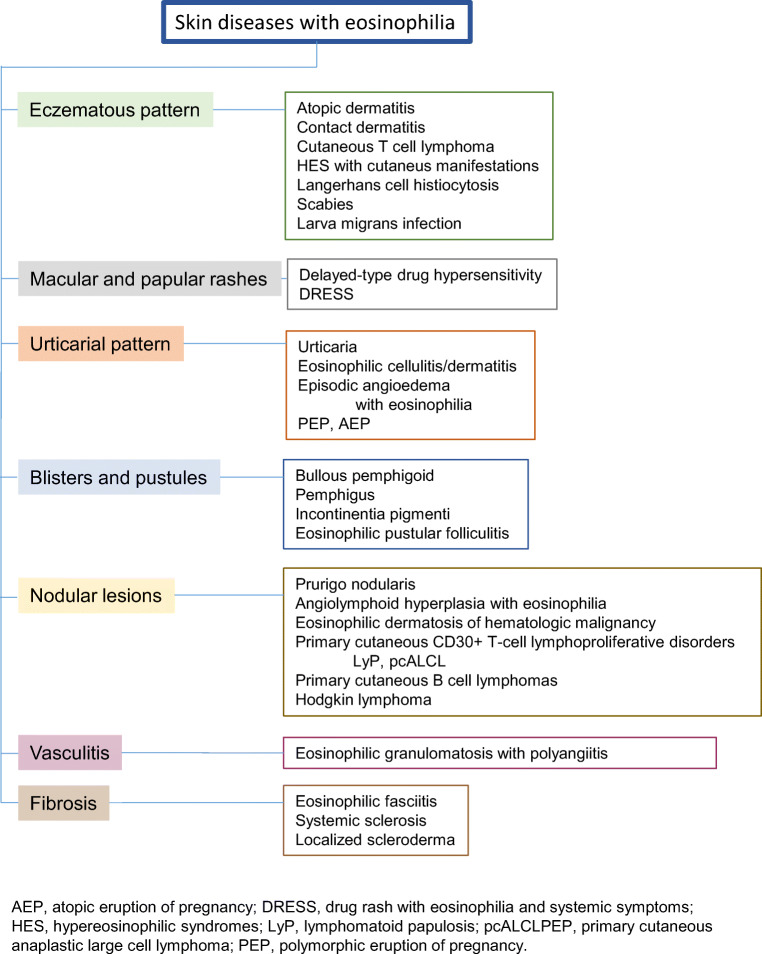
Clinical patterns of skin diseases with eosinophilia and examples

Next, we will present the spectrum of eosinophilic dermatoses based on their clinical presentations (Fig. 2) and provide current knowledge on the pathogenesis with special focus on a role of eosinophils.

### Eczematous pattern

**Fig. 3:**
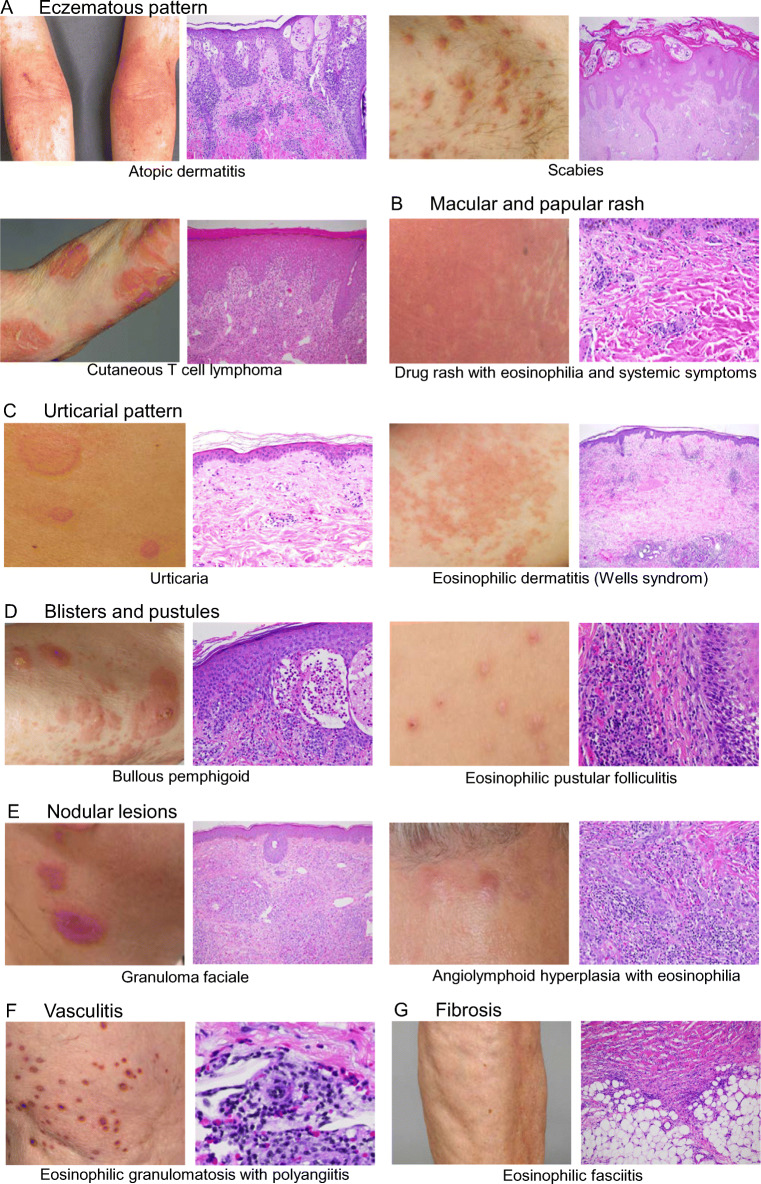
Clinical and histological presentations of dermatoses associated with eosinophilia. Images demonstrate typical skin lesions and pathological findings in H&E stained skin specimens of selected dermatoses with eczematous pattern (**A**), macular and papular rashes (**B**), urticarial pattern (**C**), blister and pustules (**D**), nodular lesions (**E**), vasculitis (**F**), and fibrosis (**G**). The key clinical and histopathological cutaneous features are the following: (**A**) Atopic dermatitis: eczema in flexural folds, spongiosis, acanthosis, superficial perivascular lymphohistiocytic infiltrate with eosinophils; scabies: linear burrows, eosinophils are main infiltrating cells; cutaneous T cell lymphoma: eczematous lesions, lymphocytic infiltrate with epidermotropism, occasional eosinophils in dermis; (**B**) DRESS: widespread erythematous macules and papules, facial edema, unspecific histology, perivascular lymphohistiocytic infiltrate with eosinophils; (**C**) urticaria: wheals, dermal edema and discrete mixed infiltrate with few eosinophils; eosinophilic cellulitis/dermatitis: urticarial patches; interstitial eosinophil infiltrate, flame figures; (**D**) bullous pemphigoid: blisters in urticarial or eczematous lesions, subepidermal blisters, numerous eosinophils in the dermis, lining at the dermal–epidermal junction and in blisters; eosinophilic pustular folliculitis: disseminated sterile pustules, intra- and perifollicular eosinophil-rich infiltrate; (**E**) granuloma faciale: brownish-red nodules and plaques; mixed, eosinophil-rich infiltrates with a grenz zone; angiolymphoid hyperplasia with eosinophilia: grouped violaceous to brownish papules and nodules, proliferation of blood vessels with epitheloid endothelial cell proliferation with cytoplasmic vacuoles, diffuse infiltrate of lymphocytes and numerous eosinophils; (**F**) esinophilic granulomatosis and polyangiitis: purpuric nodules, intra- and perivascular infiltrate of lymphocytes and eosinophils, destruction of vessel wall, fibrinoid deposition; (**G**) eosinophilic fasciitis: plate-like hardness of distal limbs, groove sign of blood vessels, thickening of fascia, infiltrate of lymphocytes, plasma cells and eosinophils in early stages

*Atopic dermatitis* is a common chronic inflammatory skin disease with a prevalence of up to 20% in children and 8% in adults [60]. It presents with highly pruritic eczematous skin lesions affecting the face, neck, and extensor sites of the extremities in infants, as well as chronically inflamed lesions with lichenification of the flexural folds in adults (Fig. 3A). The pathogenesis is based on a genetic predisposition that determines both skin barrier dysfunction and predominant type 2 inflammation, as well as environmental factors [60]. Owing to the impaired skin barrier, the skin microbiome is altered resulting in a colonization of *Staphylococcus aureus* that further harm the barrier, the susceptibility to viral infection is increased, and environmental allergens may enter in the skin. In consequence, innate and adaptive immune responses are activated. Tissue eosinophilia that has been observed in both acute and chronic AD lesions, is often accompanied by elevated blood eosinophil levels correlating with disease severity [61, 62]. In addition to intact eosinophils, cytolytic eosinophils, extracellular granules, and eosinophil toxic protein deposits have been found in AD skin [26, 61, 63, 64]. Thymic stromal lymphopoietin (TSLP) that is highly expressed by keratinocytes in AD was shown to stimulate EET formation [8]. EETs were detectable in atopy patch test reactions as model of an acute AD flare [5]. Eosinophils express type 2 cytokines and thus may actively contribute to AD inflammation. Pruritus can be stimulated by eosinophils as they release toxic granule proteins, neuromediators, and cytokines, in particular IL-31 [51]. Blocking the IL-31 receptor results in a rapid and significant reduction of pruritus followed by an improvement of AD severity [65]. As in allergic/reactive skin diseases, eosinophils express IL-11, IL-13, TGF-β, and CCL11, they would be able to induce fibrosis [4]. Thus, the following roles of eosinophils in the pathogenesis of AD can be hypothesized: defending invading pathogens by toxic granule proteins and EETs, stimulating inflammation, contributing to fibrosis, and initiating pruritus. In patients with AD, blocking IL-5 with mepolizumab had only slight effects on disease severity [66, 67]. Although mepolizumab significantly reduced blood eosinophils, it remains unclear whether it sufficiently affected eosinophil infiltration and activation in lesional AD skin.

*Contact dermatitis*, both allergic (ACD) and irritant (ICD), presents with acute and/or chronic eczematous lesions triggered by contact allergens and irritants, respectively. Whereas in ICD the innate immune system is activated, in ACD both innate and adaptive, mainly type 1, immune responses are involved. In both ICD and ACD, skin barrier disruption followed by a proinflammatory cytokine milieu and inflammation play an important pathogenic role [68]. Among skin infiltrating cells, few eosinophils are present. Eosinophils generating EETs have been detected in ACD [5]. Moreover, eosinophils that constitutively bear the lL-17 receptor together with Th17 cells are present in eczematous skin lesions. The number of Th17 cells correlated with that of MMP-9+ eosinophils, as well as tenascin deposition [69]. These observations suggest a role of eosinophils in pathogen defense and in remodeling.

Worldwide, parasitic infections are the most frequent causes of secondary eosinophilia. *Scabies and larva migrans infection* are common diseases. The skin infected by the scabies mite *Sarcoptes scabiei* presents with papules and linear burrows (Fig. 3A). It is infiltrated by mainly eosinophils, T cells, monocytes, macrophages, and mast cells, resembling a chronic allergic reaction [70]. Eosinophils have been demonstrated to generate EETs and release toxic granule proteins in both scabies and larva migrans skin lesions and thus would have the capacity to contribute to clearing of pathogens [5]. Epithelial barrier defects and epidermal proliferation similar to those in AD and psoriasis have been observed in scabies [70]. Eosinophils release cytokines and thus may stimulate adaptive, e.g., Th1, Th2, Th17, and regulatory T cells; immune responses that can be either pro- or antiinflammatory [70]. It is likely, that eosinophils contribute to bullous scabies reported in several case reports [71]. Moreover, the intense pruritus associated with scabies and larva migrans infection might be promoted by eosinophils [10].

Epidermotropic forms of *cutaneous T cell lymphoma* (CTCL) are characterized by the infiltration of malignant, clonally expanded T cells [72]. The malignant cells resemble central memory T cells in Sezary syndrome (SS), and effector memory T cells in mycosis fungoides (MF) [73]. Erythroderma, severe pruritus, atypical T cells in the peripheral blood and lymphadenopathy are typical signs of SS. MF presents with highly pruritic erythematous patches, plaques, and tumors on the skin (Fig. 3A) [74]. MF and SS have been associated with a Th 2 cytokine milieu including elevated IL-5 expression leading to eosinophilia in skin and peripheral blood [75]. Interestingly, blood eosinophilia was associated with disease progression and disease-specific death [76].

Cutaneous manifestations are the most common organ manifestations in patients with *hypereosinophilia* [77]. In patients with hypereosinophilia due to T cells with an aberrant immunophenotype, defined as lymphocytic variant of HES (L-HES), a broad spectrum of pruritic skin lesions including erythroderma, erythematous papules, urticarial plaques, and poikiloderma can be observed [78, 79]. On histology, perivascular infiltrates with lymphocytes and eosinophils with various degrees of epidermal involvement are evident [79]. The increased production of eosinophils and accumulation in the skin is due to the production of IL-5 and/or IL-3 by aberrant T cells [78, 79]. Patients with L-HES and cutaneous involvement respond usually well to anti-IL-5 and anti-IL-5 receptor therapy [14, 15].

*Langerhans cell (LHC) histiocytosis* may occur at any age but primarily affects children. The skin lesions often present with erythematous scaly or crusted papules and patches resembling seborrheic eczema. In addition, pustules, vesicles, petechiae, and purpura, as well as localized yellowish plaques and macules, are observed [80]. The infiltrate in the papillary dermis and epidermis harbors CD1a- and CD207-positive LHC and numerus eosinophils [80]. Cytokines that may directly affect eosinophils, are produced by either LHC, e.g., GM-CSF, IL-10, IFN-γ, or T cells and macrophages, e.g., IL-3, IL-2, IL-4, IL-5, and TNF-α [81, 82].

### Macular and papular rashes

*Delayed-type drug hypersensitivity reactions* (DDH) are considered to be mainly T cell mediated immune responses [83]. Eosinophils are commonly found both in the blood and, to a smaller extent, in lesional skin of patients with DDH [84]. Despite this finding, surprisingly little is known about the pathophysiological role of eosinophils in DDH.

Most DDH have a benign disease course such as maculopapular drug eruptions [84]. Other, rare benign DDH are the fixed drug eruption and the symmetrical drug-related intertriginous and flexural exanthema (SDRIFE). Severe DDH are rare, but potentially life-threatening. They include acute general exanthematous pustulosis (AGEP), the hallmark of which are nonfollicular pustules on an erythematous ground and concurrent systemic involvement in about 20% of cases [85], Stevens–Johnsons syndrome and toxic epidermal necrolysis that are characterized by epidermal detachment and potential mucosal involvement, and drug rash with eosinophilia and systemic symptoms (DRESS), an eosinophil-predominated reaction [86].

*DRESS*, also known as drug-induced hypersensitivity syndrome, has an estimated incidence of 10 cases per million per year [86]. The diagnosis of DRESS is based on a combination of clinical symptoms and laboratory parameters (Table 1) [87, 88]. Cutaneous signs are rather nonspecific: a maculopapular rash is the most common presentation, but urticarial lesions and mucosal involvement have also been reported [86, 87, 89] (Fig. 3B). Additional signs of DRESS include facial edema, enlarged lymph nodes and fever, as well as positive serology for viruses such as human herpes virus 6 (HHV6) and Epstein–Barr virus (EBV) [90]. These features are paralleled by a dysfunction of potentially any organ (Table 1). The liver is most commonly affected (ca. 75% of the cases), followed by kidneys and lungs (ca. 35%) [90, 91]. The mortality rate of DRESS is 5–10% [92]. There is no pathognomonic histopathology of DRESS, but interface dermatitis is the most common finding [50, 93]. While blood eosinophilia is a key feature of DRESS and present in the vast majority of patients, cutaneous eosinophil infiltration and the presence of atypical lymphocytes have been reported less frequently [50, 89].

**Table 1:** Diagnostic criteria of DRESS (RegiSCAR) [87]

Criteria	No	Yes	Unknown
Fever >38.5°C	−1	0	−1
Lymphadenopathy	0	1	0
Skin rash >50% body surface Edema, infiltration, scaling	0 −1	1 1	
Resolution in >15 days	−1	0	−1
Atypical lymphocytes	0	1	0
Blood eosinophilia 10–19.9% or 700–1499 Gpt/l >20% or >1500 Gpt/l	0	1 2	
Evaluation of other potential causes (antinuclear antibodies, blood culture, hepatitis A, B, C serology, chlamydia/mycoplasma)	0	1	0

*Heart, liver, kidney, muscle, pancreas or other

Scores 1–3: diagnosis uncertain; score 4 or higher: diagnosis certain

In DRESS, T cells recognizing drug-derived haptens expand, a process that can be associated with reactivation of certain viruses such as HHV6, herpes virus 1, EBV and cytomegalovirus [88]. IL-5 is considered the main driver of eosinophil expansion and activation leading to organ damage and dysfunction in DRESS [94]. The efficacy of antibodies targeting the IL-5 / IL-5 receptor axis in severe DRESS are an evidence for the key role of eosinophils in the pathogenesis [95–97].

### Urticarial pattern

In *urticaria* that presents with wheals, angioedema or both, mast cells are the key drivers [98]. The acute form, clearing within 6 weeks, is very common and mainly associated with infections of the upper respiratory tract, drugs, and IgE-mediated allergies. Physical stimuli such as heat, cold, vibration, and UV light are among the triggers of chronic inducible urticaria. In a subgroup of patients with chronic spontaneous urticaria (CSU), autoimmune responses have been identified as relevant pathogenic mechanisms [98]. Eosinophils are found among the mixed inflammatory infiltrate in the dermis (Fig. 3C). Since eosinophils express tissue factor and VEGF, as well as leukotrienes, they may increase vascular permeability resulting in dermal edema and wheal formation [99–101]. A recent review pointed to eosinophil-mast cell interaction: eosinophils may directly stimulate mast cells via the release of toxic granule proteins provoking degranulation and stem cell factor [101]. Interestingly, blood eosinopenia is associated with higher disease activity and poor response to treatment in CSU patients, in particular those with type IIb autoimmune CSU [102].

*Eosinophilic cellulitis/dermatitis* (EC), also referred to as Wells’ syndrome, is a rare dermatological condition characterized by persistent urticarial plaques and sometimes blister formation, as well as an eosinophil infiltrate in the dermis (Fig. 3C) [103]. Blood eosinophilia may be present. EC has been considered an organ restricted eosinophilic disorder among hypereosinophilic syndromes [104]. An increased expression of IL-5 has been detected in both tissue and blood [105]. Both eosinophils scattered throughout the dermis and extracellular eosinophil granules have been observed [4, 5]. EET formation is prominent and accentuated in eosinophil clusters, where up to 50% of eosinophils generate EETs [5]. A typical, but not pathognomonic finding is the presence of flame figures that correspond to amorphous collagen fibers covered by eosinophil granule proteins suggesting a pathogenic role of toxic granule proteins [5, 106]. Recently, an expression of CD25, the alpha-chain of the IL-2 receptor, on blood and tissue eosinophils of patients with EC that is mediated by high IL-5 expression, was demonstrated. Thus, IL-2 can act as potent priming factor, resulting in a strong ECP release upon additional stimulation with platelet activating factor [11].

Recent data emphasize high effectiveness of mepolizumab, an anti-IL-5-antibody, in the treatment of EC [107].

*Episodic angioedema with eosinophilia* (EAE), first described by Gleich et al. 1984, is a rare episodic variant of hypereosinophilic syndromes. It presents with recurrent attacks of angioedema, urticaria, transient weight gain, fever, and elevated leukocyte counts [108]. Abnormal CD3- CD4+ T cells have been detected in patients with EAE [109, 110]. Interestingly, a cycling of eosinophils together with other cell lineages including neutrophils and lymphocytes has been observed [109]. In addition to corticosteroids, mepolizumab and imatinib have successfully been applied in patients with EAE [111, 112].

Among specific dermatoses of pregnancy, *polymorphic eruption of pregnancy* (PEP) and *atopic eruption of pregnancy* (AEP) are the most frequent ones. They are associated with intense pruritus [113]. Clinically, AEP patients present with eczematous lesions and/or prurigo nodules starting in the second or third trimester. PEP typically affects primigravidae showing pruritic urticarial papules and plaques [113]. The histopathological analysis of skin specimens revealed the presence of eosinophils in 14/19 cases of AEP and in all 22 cases of PEP [113].

### Blisters and pustules

*Bullous pemphigoid* is the most common autoimmune bullous skin disease affecting mainly the elderly. It is characterized by an autoimmune response against hemidesmosomal antigens, involving autoreactive T and B cells. BP presents with urticarial and eczematous lesions, as well as in general blisters. Eosinophils infiltrating the skin are a striking histopathological finding. They are located in the dermis, where they often line along the dermal-epidermal junction, and in blisters (Fig. 3D). Cytokines known to recruit and activate eosinophils such as IL-5 and eotaxin are expressed in lesional skin [114, 115]. In the presence of BP antibodies, IL-5-activated eosinophils have been demonstrated to cause splitting at the dermal-epidermal junction in an ex vivo skin model [6]. BP is associated with severe pruritus. Elevated IL-31 serum levels correlating with numbers of eosinophil, which are a source of IL-31, have been found in BP patients [10, 116]. In a clinical trial, the anti-IL-5 antibody mepolizumab failed to significantly improve BP [117]. The lack of efficacy could be explained by an insufficient reduction of tissue eosinophils [117].

The *pemphigus* group is characterized by acantholysis of keratinocytes due to antidesmoglein and antidesmocolin antibodies resulting in blisters and erosions. Pemphigus vegetans has been associated with eosinophil infiltration in the skin, specifically eosinophil exocytosis and eosinophilic abscesses, as well as elevated blood eosinophil and ECP levels [118]. However, the role of eosinophils in terms of epidermal disintegration or hyperproliferation remains unclear.

*Incontinentia pigmenti* (IP) is caused by mutations of an X-linked regulatory gene, termed nuclear factor-kappa B (NF-κB) essential modulator (NEMO). The combination of eosinophilic spongiosis, vesiculation, and dyskeratotic keratinocytes is a distinct feature of IP [119]. In the initial, vesiculo-bullous stage, the epidermis expresses eotaxin correlating with the accumulation of eosinophils [120]. To note, the eotaxin promoter has an NF-κB binding site [121].

*Eosinophilic pustular folliculitis* (EPF) is an inflammatory skin conditions, typically presenting with sterile follicular papulopustules (Fig. 3D). Three different subtypes have been described so far, classical (Ofuji disease), immunodeficiency-associated and EPF of infancy. The prompt therapeutic response to indomethacin is pathognomonic. The histology shows an accumulation of eosinophils in the pilosebaceous unit. Recently, an increased expression of prostaglandin D synthetase by inflammatory cells including eosinophils has been demonstrated around the pilosebaceous unit [122]. Prostaglandin D2 induces mRNA expression of eotaxin-3 (CCL26), a potent chemoattractant for eosinophils, by human sebocytes in a dose-dependent manner. These findings might explain both the inflammatory pattern, as well as the therapeutic effect of indomethacin [122]. In addition, increased expression of TNF-α by tissue eosinophils has been found in EPF [4]. A pathogenic role of eosinophils is underlined by the fact that the anti-IL-5 receptor antibody benralizumab is effective in EPF [123].

### Nodular lesions

*Prurigo nodularis* stands for a clinical reaction pattern induced by chronic pruritus and permanent scratching presenting with symmetrically distributed, firm, hyperkeratotic, excoriated nodules [124]. On histology, few eosinophils have been detected in all specimens investigated, and extracellular deposition of the eosinophil granule proteins were found in most of them [125]. As eosinophils produce an armory of toxic proteins, cytokines and mediators that can activate nerve cells, they are predisposed to contribute to pruritus [126].

Solitary asymptomatic brown-red to livid papules, nodules or plaques on the face are the typical clinical manifestation of *granuloma faciale* (EF). Histologically, it is characterized by a mixed cellular infiltrate, composed of lymphocytes, neutrophils, and eosinophils (Fig. 3E). An infiltrate-free grenz zone in the papillary dermis is pathognomonic. Recently, a clonal expansion of skin-homing CD4+ T cells has been demonstrated alongside with an increase in local IL-5 expression [127]. Increased IL-5 expression is associated with both increased eosinophil recruitment, survival, and activation. The role of the eosinophil itself in the pathogenesis of granuloma faciale has not been determined yet. Recently, an EF sharing features with an IgG4-associated sclerosing disease has been reported [128].

*Angiolymphoid hyperplasia* with eosinophilia (ALHE) is a benign vascular neoplasia with an eosinophil-rich infiltrate, presenting as livid papule or plaque on the face. Kimura disease is defined by deep subcutaneous swellings, the presence of blood eosinophilia, increased levels of total IgE alongside with a lymphadenopathy [129]. Currently Kimura disease and ALHE are considered as spectrum of the same disease. The infiltrate is composed of T cells, B cells, eosinophils, and plasma cells (Fig. 3E).

In ALHE lesions, eosinophils show a strong expression of IL-13 and TGF-β , as well as CCL24 and MMP-9, pointing toward a role of the eosinophil in immunoregulation and tissue remodeling [4]. Mast cells and eosinophils in close proximity, as well as T cells, can be found in Kimura disease [130]. As mast cells and eosinophils produce IL-5, CCR3 and RANTES, a bidirectional interaction of eosinophils und mast cells can be assumed [130, 131].

*Eosinophilic dermatosis of hematologic malignancy* (EDHM), previously also referred to as exaggerated insect bite-like reaction, is mainly associated with chronic lymphatic leukemia [132]. Clinically, it presents with pruritic papules and occasionally bullae. Based on a clinical and immunohistochemical study of six EDHM cases, Th 2 cells reactive to malignant B cells by producing IL-5 have been suggested to be responsible for eosinophil accumulation and activation, and subsequent clinical signs and symptoms [133]. There is evidence that neoplastic B cells are part of the infiltrate in EDHM lesions [134].

Eosinophil infiltration is observed in various *cutaneous lymphomas*. Primary cutaneous CD30+ T-cell lymphoproliferative disorders include lymphomatoid papulosis (LyP) and anaplastic large cell lymphoma (pcALCL) that manifest with papules, plaques, and nodules [135]. Patients with LyP that is self-healing, have an increased risk of developing secondary lymphoproliferative disorders such as a CD30+ ALCL, Hodgkin lymphoma or mycosis fungoides [135]. The presence of eosinophils in skin lesions is due to IL-3, IL-5, or GM-CSF expression by the malignant T cells [136]. In primary cutaneous B cell lymphomas, an aberrant expression of the B cell lymphoma (BCL)-6 transcriptional repressor protein regulating the transcription factor GATA-3 is supposed to promote type 2 inflammation and eosinophilia [137]. In *Hodgkin lymphoma*, Hodgkin/Reed Sternberg tumor cells produce TNF-α that stimulates eotaxin production by fibroblasts leading to eosinophil recruitment [138]. Tissue eosinophilia has been associated with poor prognosis [12]. Eosinophils secrete CD30 ligand that via binding to CD30 on Hodgkin cells may promote tumor cell proliferation and survival [12].

Opposite to the protumorigenic functions, eosinophils have been reported to exert antitumorigenic activity. In melanoma patients, the response to immune checkpoint inhibitor therapy correlates with the increase of peripheral blood eosinophil counts [13, 139]. Moreover, eosinophil activation and degranulation of MBP, as well as an association of eosinophil and CD8+ T cells numbers infiltrating the tumor tissue, have been observed, suggesting both direct and indirect effects of eosinophils in tumor defense [13].

### Vasculitis

The diagnosis of *eosinophilic granulomatosis* with *polyangiitis* (EGPA) is based on the occurrence of asthma and other allergy symptoms, eosinophilia in blood and tissues followed by eosinophilic granulomas and vasculitis that may affect all organs including skin in the third phase (Fig. 3F) [140]. As potential roles of eosinophils in EGPA pathogenesis, the production of cytokines such as IL-25 shaping Th2 inflammation, degranulation, toxic effects on tissues leading to necrotizing granulomas, and destruction of small to medium-sized vessel walls, as well as the release of tissue factor and activation of platelets contributing to thromboembolism, have been discussed [140]. Antibodies directed against IL-5 or IL-5 receptor have been shown to be effective in EGPA [141–143].

### Fibrosis

*Eosinophilic fasciitis* (EF) often starts with an edematous phase followed by a bilateral, symmetrical solid hardening of the skin on the limbs why it has been considered a subtype of scleroderma (Fig. 3G) [144]. Blood and tissue eosinophilia occur during the early stages. In addition, a thickened fascia, as well as an inflammatory infiltrate of lymphocytes and plasma cells, occasionally a fibrosis of the dermis can be observed on histology [144]. In patients with EF, elevated IL-5 and ECP levels have been detected [144, 145]. As eosinophils are known to stimulate fibrosis via the release of toxic granule proteins, metalloproteinases, and TGF-β, they are assumed to contribute to the pathogenesis of EF.

Eosinophils are part of the cutaneous inflammatory infiltrate in *systemic sclerosis and localized scleroderma* [146]. For the latter, an endothelial injury followed by the recruitment of eosinophils, T cells, and macrophages, as well as the secretion of fibrogenic cytokines including IL-4, IL-6, and TGF-β, have been considered as important pathogenic factors [146].

## Targeting eosinophils as therapeutic approach

Over the last decade with the development of targeted therapies, eosinophils have caught attention as treatment targets. Antibodies blocking IL-5 and IL-5 receptor have been approved for asthma and have successfully been tested in hypereosinophilic syndromes. An increasing number of case reports and case series demonstrate the efficacy of mepolizumab, reslizumab, and benralizumab in various eosinophilic dermatoses (Table 2). By reducing type 2 inflammation and thus impacting eosinophil responses, drugs such as dupilumab are effective in many diseases beyond AD

**Table 2:** Antieosinophil targeted therapies

Target	Name	Effect on eosinophils	Effective in
IL-5	Mepolizumab	Production, survival, recruitment, activation	HES with eosinophilic dermatitis [14] EGPA [141] DRESS [95] Eosinophilic cellulitis [107] Kimura disease [147] Urticaria [148]
	Reslizumab		Chronic spontaneous urticaria, cold urticaria [149] EGPA [143]
IL-5Rα	Benralizumab	Survival, recruitment, activation	HES [15] DRESS [97] EGPA [142] EPF [123]
Eotaxin-1	Bertilimumab	Recruitment	BP Phase 2a Study (NCT02226146), results not available
Siglec-8	Antolimab	Depletion + reduction of mast cell activation	Chronic spontaneous urticaria [101, 150]
IL-4/IL-13Rα	Dupilumab	Activation + decrease of type 2 inflammation	AD [151] BP [152] EDHM [153, 154]
IL-13	Tralokinumab Lebrikizumab	Activation + IL-13 production	AD [155, 156]
Janus kinase	Tofacitinib Ruxolitinib	Blocking JAK and STAT3 + decrease type 2 inflammation	HES with cutaneous involvement [157]
	Baricitinb Upadacitinib Abrocitinib		AD [158–160]

*AD*, atopic dermatitis; *BP*, bullous pemphigoid; *DRESS*, drug rush with eosinophilia and systemic symptoms; *EDHM*, eosinophilic dermatosis of hematologic malignancy; *EGPA*, eosinophilic granulomatosis and polyangiitis; *EPF*, eosinophilic pustular folliculitis; *HES*, hypereosinophilic syndrome

## Conclusion

Eosinophil infiltration in the skin is a frequent finding in a broad spectrum of dermatoses and often an indicative finding for the diagnosis. However, the exact roles of eosinophils need to be defined in most of these diseases. The use of novel targeted therapies and monitoring their effects on clinical signs and symptoms, as well as the numbers and functions of eosinophils including their interactions with tissue specific and inflammatory cells, provide an opportunity to better define the pathogenic role of eosinophils.

## Data Availability

Not applicable.
